# Anti-Müllerian hormone is correlated with cumulative live birth in minimal ovarian stimulation with clomiphene citrate: a retrospective cohort study

**DOI:** 10.1186/s12884-020-03446-1

**Published:** 2020-11-27

**Authors:** Kenji Ezoe, Xiaowen Ni, Tamotsu Kobayashi, Keiichi Kato

**Affiliations:** Kato Ladies Clinic, 7-20-3 Nishishinjuku, Shinjuku-ku, Tokyo, 160-0023 Japan

**Keywords:** Anti-Müllerian hormone, Blastocyst formation, Clomiphene citrate, Cumulative live birth, Ovarian response

## Abstract

**Background:**

Several studies have investigated the correlation between the serum anti-Müllerian hormone (AMH) level and in vitro fertilization (IVF) outcomes in controlled ovarian stimulation cycles; however, studies regarding the correlation of the serum AMH level with IVF outcomes in minimal ovarian stimulation cycles remain limited. In this study, we aimed to analyze the correlation of the serum AMH level with ovarian responsiveness, embryonic outcomes, and cumulative live birth rates in clomiphene citrate (CC)-based minimal ovarian stimulation cycles.

**Methods:**

Clinical records of 689 women whose entire ovarian stimulation regimen consisted solely of minimal stimulation cycle IVF using CC alone from November 2017 to October 2019 were retrospectively reviewed. The association between IVF outcomes and the serum AMH level before the initiation of the first fertility treatment was analyzed. Furthermore, the correlation of the serum AMH level with cumulative live birth rates after IVF treatment was assessed. The Cochran-Armitage test, Pearson’s chi-squared test, Spearman rank correlation test, Student’s t-test, one-way analysis of variance, logistic regression analysis, Kaplan-Meier method and Cox proportional hazards model were used to analyze the data.

**Results:**

The serum AMH level positively correlated with the number of retrieved oocytes, blastocyst formation rate, blastocyst cryopreservation rate, and live birth rate per oocyte retrieval in CC-based minimal ovarian stimulation cycles without any exogenous gonadotropin administration. Furthermore, the cumulative live birth rate and treatment period required for conceiving were strongly associated with the serum AMH level at the initiation of fertility treatment.

**Conclusions:**

A low serum AMH level correlated with low ovarian responsiveness, impaired pre-implantation embryonic development, and decreased cumulative live birth rate in CC-based minimal ovarian stimulation cycles. Therefore, the cycle success rate would be predicted by measuring the serum AMH level in minimal ovarian stimulation with CC alone.

**Supplementary Information:**

**Supplementary information** accompanies this paper at 10.1186/s12884-020-03446-1.

## Background

In women, anti-Müllerian hormone (AMH) is synthesized by granulosa cells in preantral and early antral stage (5–8 mm diameter) follicles [1, 2]. AMH regulates follicular development by inhibiting the initial recruitment from the primordial pool [3, 4]. Since AMH produced in the ovary is secreted into the circulation, it can be measured in the serum. Some studies reported that the serum AMH level remained stable throughout the menstrual cycle [5–8]. In addition, the serum AMH level strongly correlated with the resting pool of ovarian follicles; therefore, AMH measurement may be used to predict the reduction in the number of growing follicles and subsequent menopause [9–11]. These AMH physiological characteristics can be applied to assisted reproductive technologies.

Previous studies reported that serum AMH level was an effective predictor of oocyte retrieval among women with infertility undergoing controlled ovarian stimulation (COS) for in vitro fertilization (IVF): that is, the serum AMH level positively correlated with the number of retrieved oocytes after the administration of exogenous gonadotropins [12–16]. Furthermore, in COS cycles, women with higher serum AMH levels have higher ongoing pregnancy rates as well as live birth rates [17, 18]. On the contrary, in the case of natural conception without any exogenous gonadotropins, a low serum AMH level is not associated with a reduced fecundability rate or a lower cumulative probability of conceiving, suggesting that the serum AMH level cannot be used as a predictor of pregnancy outcomes in spontaneous natural cycles without gonadotropin [19].

Clomiphene citrate (CC) is often administered alone in the minimal ovarian stimulation protocol for IVF [20–23]. CC binds hypothalamic estrogen receptors and induces the secretion of gonadotropin-releasing hormone (GnRH) by altering the negative feedback effect of estrogen on the hypothalamus, resulting in the promotion of endogenous gonadotropin secretion. Minimal ovarian stimulation protocols can decrease the dose and duration of exogenous gonadotropin administration, thereby reducing the influence on the ovaries and reducing patient distress and complications [24–26]. In addition, IVF outcomes in minimal ovarian stimulation protocols are considered similar to those of COS cycles, especially in patients who are poor responders [20, 27–29]. Although the serum AMH level is predictive of ovarian response and IVF outcomes after gonadotropin administration, the predictive ability of the serum AMH level in clomiphene-based minimal stimulation cycles is still unknown. Therefore, to reveal whether the serum AMH level can be used as a predictor for IVF success in minimal stimulation cycles, we aimed to retrospectively analyze the correlation of the serum AMH level with ovarian responsiveness, embryonic outcomes, and cumulative live birth rates in CC-based minimal ovarian stimulation.

## Methods

### Patients

From November 2017 to June 2018, 1132 patients were scheduled to undergo their first oocyte retrieval cycle at our center. Patients consulted with their gynecologists to decide their treatment plan based on their preference or menstrual cycle: minimal stimulation cycle or natural cycle IVF. Patients who underwent natural cycle (*n* = 66), CC-based minimal stimulation cycle with gonadotropin administration (*n* = 42), CC-based and minimal stimulation cycle with letrozole (*n* = 42), or several types of stimulation cycles (*n* = 240) of their IVF treatments during the study period were excluded. Thus, a total of 689 women whose entire ovarian stimulation regimen was solely minimal stimulation cycle IVF using CC were included (Supplemental Table 1). The serum AMH level was measured on day 3 of the first IVF treatment using Elecsys® AMH (Roche Diagnostics, Rotkreuz, Switzerland). The clinical records on ovarian responsiveness, embryonic outcomes, and cumulative live birth rates of the included patients were retrospectively reviewed.

### Minimal ovarian stimulation cycle IVF

The minimal stimulation protocol with CC was previously reported [30, 31]. Briefly, CC (Fuji Pharma Co., Ltd., Tokyo, Japan) was administered with an extended regimen from cycle day 3 until induction of final oocyte maturation. Monitoring began on day 8–10 and included an ultrasound scan and hormonal profiles (estradiol, luteinizing hormone (LH), and progesterone), and performed every other day until the ovulation triggering day. When the leading follicle reached 18 mm with a concomitant estradiol level ≥ 250 pg/ml, ovulation was triggered using buserelin (Suprecur; Mochida Pharmaceutical Co., Ltd., Tokyo, Japan or Buserecur; Fuji Pharma Co., Ltd.). Oocyte retrieval was performed 34 to 35 h after stimulation using a 21-G needle (Kitazato Corporation, Shizuoka, Japan). To evaluate the efficiency of follicle aspiration, the oocyte collection rate was calculated as the number of COCs divided by the number of aspirated follicles.

The collected cumulus-oocyte complexes (COCs) were washed and transferred to human tubal fluid (HTF) medium (Kitazato Corporation) with paraffin oil at 37 °C (gas phase: 5% O_2_, 5% CO_2_, and 90% N_2_) for culturing, until either conventional IVF (cIVF) was performed 3 h later, or in cases of intracytoplasmic sperm injection (ICSI), denudation was performed 4 h after oocyte retrieval [32, 33]. For ICSI, cumulus cells were removed, and the denuded oocytes were cultured in HTF medium for 1 h before ICSI.

Sperm samples were collected by masturbation and washed by centrifugation through 70 and 90% density gradients (Isolate; Irvine Scientific, Santa Ana, CA, USA). Prepared sperm was cultured in HTF medium at 37 °C (gas phase: 5% CO_2_ and 90% N_2_) until use.

### Conventional insemination, ICSI, embryo culture, and cryopreservation

For patients undergoing cIVF, 10% of serum substitute (Irvine Scientific) supplemented HTF medium was used as a fertilization medium [32]. COCs were cultured with sperm (100,000 sperm/mL) at 37 °C (gas phase: 5% CO_2_ and 90% N_2_). After the confirmation of second polar body extrusion at 5 h after insemination (day 0), the fertilized oocytes were individually cultured in medium drops (ONESTEP medium; Nakamedical, Inc., Tokyo, Japan) with paraffin oil. For patients undergoing ICSI, oocytes were immediately placed in ONESTEP medium after sperm injection [32, 33]. All embryos were cultured at 37 °C (gas phase: 5% O_2_, 6% CO_2_, and 89% N_2_). Cleavage-stage embryos were graded using Veeck’s criteria at 42 h post-insemination [34], and blastocyst quality was evaluated according to Gardner’s criteria [35]. The embryos were transferred on day 2 or cultured until the cleavage or blastocyst stage and then vitrified for subsequent use in embryo transfer cycles. Embryo vitrification was performed using Cryotop® (Kitazato Biopharma, Japan), as described previously [36]. The blastocyst cryopreservation rate was calculated as the number of cryopreserved blastocysts divided by the number of cleaved embryos cultured to the blastocyst stage.

### Embryo transfer

Single fresh or vitrified-warmed embryo transfers were performed in natural cycles [33, 37–39]. Briefly, the transfers of cleavage stage embryos and blastocysts were performed on days 2–3 and day 5 after oocyte retrieval or with the confirmation of ovulation, respectively. Dydrogesterone (30 mg/day) was administered orally after the transfer procedure. In addition, in cases with insufficient luteal function, progesterone was administered intravaginally (Lutinus, Ferring Pharmaceuticals, Saint Prex, Switzerland) until 9th week of pregnancy. The clinical pregnancy rate and ongoing pregnancy rate were defined by ultrasonographic observation of the gestational sac at 5–6 weeks after embryo transfer and observation of fetal heartbeats at 7 weeks after embryo transfer, respectively.

### Statistical analyses

All statistical analyses were performed using JMP software (SAS Institute Inc., Cary, NC, USA). The Spearman rank correlation test was used to measure the degree of association between two continuous variables. The proportion data were analyzed using the Pearson’s chi-squared tests. Continuous parameters were compared using Student’s *t*-test or one-way analysis of variance, with significance evaluated using Tukey’s test for post-hoc analysis. The univariate logistic regression analysis was used to identify the covariates that were potentially associated with the outcomes. The likelihood ratio test for the significance of the coefficient was performed, and the variables with *P* <  0.05 were used as confounders. Similarly, multivariate logistic regression analysis for theoutcomes was used to adjust the bias (using the confounders) and verify the statistical significance (using Wald statistic). Odds ratios and adjusted odds ratios are reported with 95% confidence intervals for each group. A receiver operating characteristic (ROC) analysis was also performed, and the area under the ROC curve and cut-off value was calculated. The cumulative live birth was analyzed using the Kaplan-Meier method and Cox proportional hazards model. A *P*-value of < 0.05 was considered statistically significant.

## Results

### Inclusion criteria

During the study period, 1132 patients underwent the first oocyte retrieval, of whom 689 patients underwent the oocyte retrieval in CC-based minimal stimulation (Supplemental Table 1). In the present study, all included patients responded to CC, exhibited the follicular development after CC administration, and underwent oocytes retrieval (100%, 689/689). In total, the successful oocyte retrieval rate (≥ 1 oocyte) was 91.1% (628/689).

### Clinical significance of the serum AMH level in CC-based minimal stimulation IVF

Results of the correlation analysis of the serum AMH level with patient and cycle characteristics using Spearman’s rank correlation coefficient are shown in Table 1. The serum AMH level was significantly associated with the patient’s age, serum follicle stimulating hormone (FSH) and LH levels on day 3, number of antral follicles on day 3, and LH level on the day of the stimulation. The serum AMH level was also correlated with the number of retrieved oocytes in CC-based minimal stimulation cycles. The oocyte collection rate was significantly associated with the serum AMH level.

**Table 1:** Correlation of serum AMH level^a^ with patient and cycle characteristics

	Spearman’s rank correlation coefficient	*P* value
Age of women	−0.3335	< 0.0001
Serum follicular stimulating hormone level on day 3	−0.4732	< 0.0001
Serum luteinizing hormone level on day 3	0.1016	0.0284
Serum estradiol level on day 3	0.0130	0.7627
Antral follicle count on day 3	0.6890	< 0.0001
Serum estradiol level on the day of the trigger	0.0524	0.1704
Serum progesterone level on the day of the trigger	−0.0573	0.1332
Serum luteinizing hormone level on the day of the trigger	0.0848	0.0263
No. of fully developed antral follicles	0.0508	0.1830
No. of the retrieved oocytes	0.1666	< 0.0001
Oocyte collection rate (/aspirated follicles)^b^	0.1506	< 0.0001

*AMH* anti-Müllerian hormone

^a^The serum AMH level was used as the continuous parameter in this analysis

^b^The oocyte collection rate was calculated as the number of cumulus-oocyte complex (COC) / number of aspirated follicles

Results of the correlation analysis between the serum AMH level and IVF outcomes using univariate logistic regression analysis are shown in Table 2. While the serum AMH level was not associated with the fertilization and cleavage rates, it was positively correlated with blastocyst formation and cryopreservation rates. Furthermore, the increase in serum AMH level significantly correlated with the improvement in cumulative rates of clinical pregnancy, ongoing pregnancy, and live birth. The ROC analysis demonstrated that the cut-off level of the serum AMH for the establishment of live birth in the first oocyte retrieval cycle was 1.42 ng/mL. These results indicated that the serum AMH level was significantly correlated with embryonic and pregnancy outcomes in CC-based minimal stimulation cycle IVF.

**Table 2:** Odds ratio of the serum AMH level^a^ for IVF outcome in the first cycle

	No. of events	Odd ratio	95% confidential interval	P value	AUC
Fertilization	1295	1.077	0.989–1.179	0.1000	0.544
Cleavage	1295	1.059	0.977–1.153	0.1770	0.537
Blastocyst formation	635	1.123	1.012–1.256	0.0337	0.566
Blastocyst cryopreservation	635	1.149	1.041–1.277	0.0074	0.581
Clinical pregnancy in the first cycles	689	1.306	1.186–1.444	< 0.0001	0.643
Ongoing pregnancy in the first cycles	689	1.321	1.200–1.460	< 0.0001	0.644
Live birth in the first cycles	689	1.319	1.198–1.457	< 0.0001	0.642

*AMH* anti-Müllerian hormone, *AUC* area under the curve, *IVF* in vitro fertilization

^a^The serum AMH level was used as the continuous parameter in this analysis

### First IVF outcomes stratified by the serum AMH level

The clinical outcomes of the first cycle were stratified by the AMH cut-off value for live birth (< 1.42 or ≥ 1.42 ng/mL). Patient characteristics are shown in Supplemental Table 2. Patients in the high AMH group were significantly younger than those in the low AMH group. The collection rate in the low AMH group (87.1%) was lower than that in the high AMH group (94.2%) (Supplemental Table 3). The number of antral follicles on day 3, fully developed antral follicles on the day of oocyte retrieval, and retrieved oocytes were higher in the high AMH group than in the low AMH group. The oocyte collection rate in the high AMH group was significantly higher than that in the low AMH group. Patients with a high serum AMH level obtained more fertilized oocytes, cleaved embryos, and blastocysts in the first oocyte retrieval. Furthermore, high blastocyst formation and cryopreservation rates were observed in the high AMH group. Although the morphological grade in the high AMH group was significantly better than that in the low AMH group on day 2, no difference was observed in the morphological grade on days 5–6.

Moreover, 62.2% of the patients in the low AMH group received embryo transfer, which was significantly lower than that in the high AMH group (74.2%) (Supplemental Table 4). Pregnancy outcomes after fresh- and frozen-cleaved embryo transfers were significantly better in the high AMH group than in the low AMH group. On the contrary, the outcomes after the frozen blastocyst transfers were comparable between the groups. The rates of clinical pregnancy, ongoing pregnancy, and live birth in the first IVF cycle were higher in the high AMH group than in the low AMH group. A similar tendency was observed when the clinical outcomes of the first cycle were stratified by the percentile of the AMH level (Supplemental Table 5).

To validate the above results, we performed a multivariate logistic regression analysis (Supplemental Table 6). The analysis demonstrated that the blastocyst cryopreservation rate was statistically associated with the serum AMH level. Although the rates after the frozen-cleaved embryo transfer and frozen blastocyst transfer were not related with the serum AMH level, the live birth rate after the fresh cleaved embryo transfers significantly correlated with the serum AMH level. Furthermore, the live birth rate in the first IVF cycle correlated with the patient’s age and serum AMH level (Table 3).

**Table 3:** Adjusted odds ratio for live birth rates in the first cycles^a^

	Adjusted odds ratio^b^	95% confidential intervals	*P* value
Female age	0.836	0.784–0.889	< 0.0001
Serum follicular stimulating hormone level on day 3	1.028	0.980–1.077	0.2446
Serum luteinizing hormone level on day 3	1.031	0.886–1.197	0.6921
Serum estradiol level on day 3	1.001	0.991–1.009	0.8270
Antral follicle count on day 3	1.015	0.967–1.064	0.5508
Serum AMH level^c^	1.207	1.034–1.417	0.0188

*AMH* anti-Müllerian hormone

^a^The clinical outcomes in 689 first cycles were used for this analysis

^b^Confounding factors: female age, male age

^c^The serum AMH level was used as the continuous parameter in this analysis

### Cumulative live birth rate stratified by the serum AMH level

**Fig. 1:**
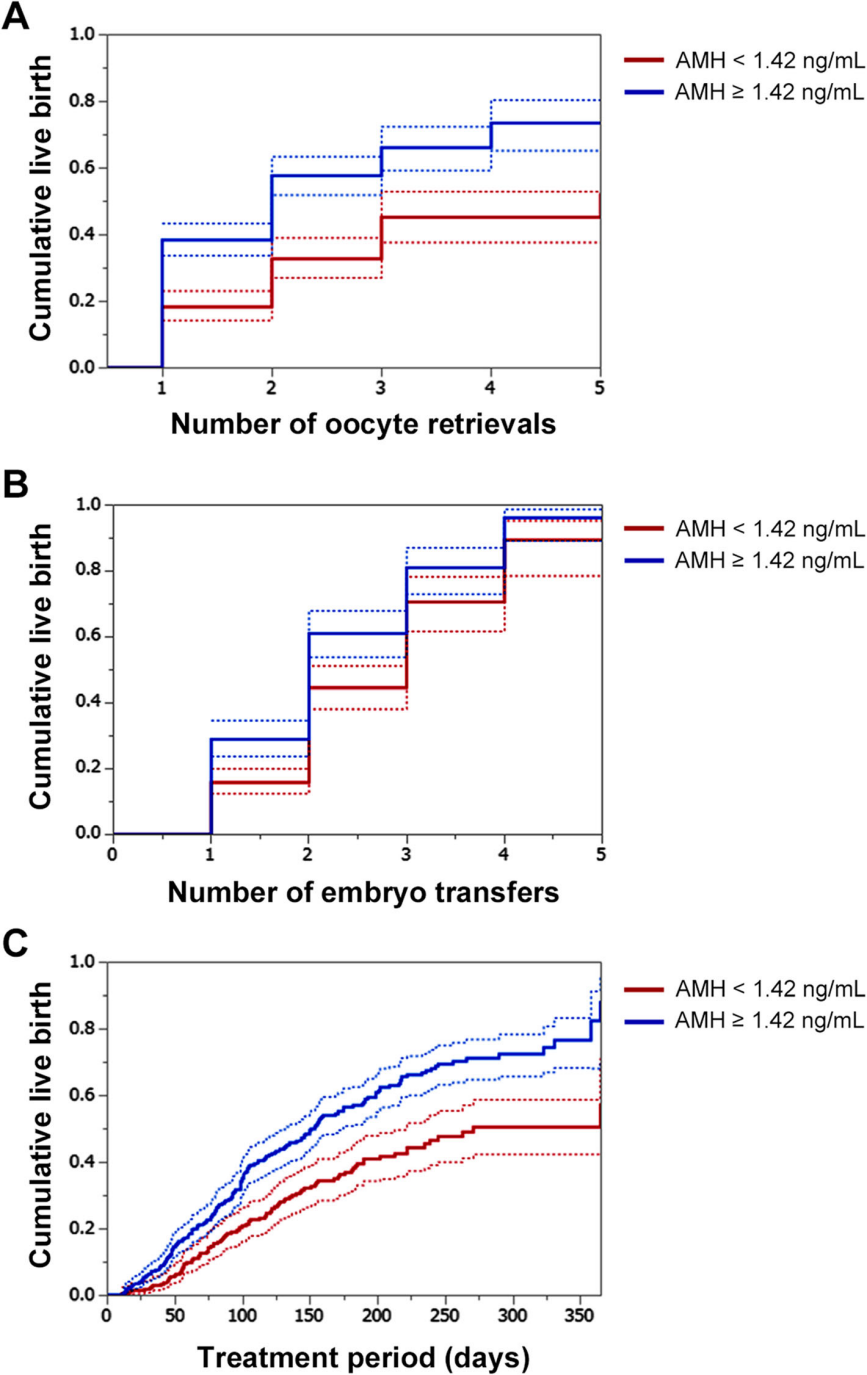
Cumulative live birth rate stratified by the serum AMH level. The estimated cumulative live birth rate per the number of oocyte retrievals (**a**), the number of embryo transfers (**b**) and treatment period (**c**). Red lines indicate the proportion of women with high serum AMH level (≥ 1.42 ng/mL), and blue lines indicate that of women with low serum AMH level (< 1.42 ng/mL). Dotted lines demonstrate the 95% confidence intervals

The crude cumulative live birth rates after the fifth oocyte retrieval in the low and high AMH groups were 33.7 and 53.2%, respectively. The estimated cumulative live birth rates after the fifth oocyte retrieval calculated using the Kaplan-Meier method were 52.6% in the low AMH group and 73.6% in the high serum AMH group (Fig. 1a). A Cox proportional hazards model demonstrated a correlation between decreased serum AMH levels and lower cumulative live birth rates (adjusted hazard ratio, 1.116; *P* = 0.0004; Table 4). The crude cumulative live birth rates after the fifth embryo transfer in the low and high AMH groups were 33.7 and 53.2%, respectively. The estimated cumulative live birth rates after the fifth embryo transfer calculated using the Kaplan-Meier method were 89.5% in the low AMH group and 96.2% in the high serum AMH group (Fig. 1b). A Cox proportional hazards model demonstrated a correlation between decreased serum AMH level and lower cumulative live birth rate (adjusted hazard ratio, 1.067; *P* = 0.0458; Table 5). We calculated the cumulative live birth rates per treatment period and found a low serum AMH level to be associated with a prolonged treatment period (Fig. 1c). The periods required for 50% live birth per total patients in the low AMH group and high AMH group were 271 and 154 days, respectively. A Cox regression analysis confirmed the positive correlation of the serum AMH level with the treatment period and successful live birth rates (adjusted hazard ratio, 1.104; *P* = 0.0015; Supplemental Table 7).

**Table 4:** Hazard ratio for cumulative live birth per the number of oocyte retrievals^a^

	Unadjusted hazard ratio (95% CI)	*P*-value	Adjusted hazard ratio (95% CI)	*P*-value
Female age	0.893 (0.867–0.921)	< 0.0001	0.895 (0.861–0.931)	< 0.0001
Male age	0.964 (0.944–0.985)	0.0006	1.010 (0.985–1.035)	0.4180
AMH^b^	1.168 (1.102–1.233)	< 0.0001	1.116 (1.049–1.184)	0.0004

*AMH* anti-Müllerian hormone, *CI* confidence interval

^a^The clinical outcomes of 689 patients were used for this analysis

^b^The serum AMH level was used as the continuous parameter in this analysis

**Table 5:** Hazard ratio for cumulative live birth per the number of embryo transfers^a^

	Unadjusted hazard ratio (95% CI)	*P*-value	Adjusted hazard ratio (95% CI)	*P*-value
Female age	0.900 (0.872–0.930)	< 0.0001	0.904 (0.861–0.931)	< 0.0001
Male age	0.967 (0.946–0.988)	0.0006	1.006 (0.985–1.035)	0.4180
AMH^b^	1.123 (1.057–1.189)	0.0001	1.067 (1.003–1.136)	0.0458

*AMH* anti-Müllerian hormone, *CI* confidence interval

^a^The clinical outcomes of 689 patients were used for this analysis

^b^The serum AMH level was used as the continuous parameter in this analysis

## Discussion

To the best of our knowledge, this is the first study demonstrating a correlation between the serum AMH level and the rates of blastocyst formation, blastocyst cryopreservation, and live birth per oocyte retrieval in CC-based minimal ovarian stimulation cycles without any exogenous gonadotropin administration. Furthermore, the present investigation showed the cumulative live birth rate and treatment period in CC-based minimal ovarian stimulation cycles to be strongly associated with the serum AMH level at fertility treatment initiation.

Some studies have investigated the correlation between serum AMH levels and IVF outcomes in COS cycles and reported that the serum AMH level can be predictive of the oocyte quality, embryo morphology, and subsequent pregnancy outcomes after exogenous gonadotropin administration [14, 40, 41]. However, studies regarding the correlation of the serum AMH level with IVF outcomes in minimal ovarian stimulation cycles remain limited. We first assessed the association between the serum AMH level and IVF outcomes in CC-based minimal ovarian stimulation cycles. As mentioned in the Introduction, serum AMH level was not associated with a cumulative probability of conceiving in cases of natural conception [18]. Normally, only one follicle is induced to develop into a Graafian follicle, and a COC is ovulated into the fallopian tube in natural conception; therefore, pregnancy outcomes in natural conception would be affected by the rest of the follicular pool as well as the serum AMH level in young women.

According to a previous study, we expected that the oocyte retrieval outcomes may not be associated with the serum AMH level in women who underwent CC-based minimal ovarian stimulation IVF as the CC-only protocol slightly promotes follicular development, and in most cases, only 1–3 COCs are ovulated. However, our data showed that the serum AMH level positively correlated with the number of retrieved oocytes even without exogenous gonadotropin administration. This AMH feature was similar to that in COS IVF cycle [12–16]. Furthermore, our results demonstrated that the decline in the serum AMH level correlated with the decrease in blastocyst formation and cryopreservation rates, but not the fertilization and cleavage rates. In line with this finding, in women with low serum AMH levels (< 1.42 ng/mL), the live birth rate after cleaved embryo transfers was significantly lower than that in women with high serum AMH levels (≥ 1.42 ng/mL), suggesting that decreased serum AMH levels are also correlated with the impaired embryo developmental competence of the oocyte and cleaved embryos to the blastocyst stage, as in COS cycles [14, 42–44].

In this study, the correlation of serum AMH levels with the live birth rates in the first cycle was also analyzed. A previous study reported that AMH is a better clinical predictor of cycle success in COS IVF cycles, although FSH, antral follicle count, and AMH are widely used to assess the ovarian reserve in women undergoing infertility evaluation [45]. Contrarily, another study demonstrated that antral follicle count was more predictive of clinical pregnancy than the serum AMH level [46]; therefore, in COS cycles, factors that best predict ovarian reserve and live birth are controversial. In CC-based minimal stimulation cycles, AMH appears to be superior to FSH and antral follicle count for the prediction of successful live birth in the first cycle. In this study, a multivariate logistic regression analysis demonstrated that the patient’s age and serum AMH level were significantly associated with the live birth rate in the first retrieval cycles, and that the FSH level and antral follicle count on day 3 were not (Table 3). Furthermore, we stratified the pregnancy outcomes by the patient’s age groups according to the Society for Assisted Reproductive Technology (SART) classification (age: young, ≤ 37; middle, 38–40; and advanced, ≥ 41 years) and the percentile of serum AMH levels (Supplemental Table 8). When the patient’s age was limited, pregnancy outcome was significantly correlated with the serum AMH level in all age groups. When the serum AMH level was limited, the patient’s aging resulted in poor pregnancy outcomes. These results confirm the previous reports that examined the association among the pregnancy outcomes, patient’s age, and serum AMH level in COS IVF cycles [47, 48]. Our results also suggest that the clinical outcomes in CC-based minimal stimulation cycles can be predicted using the patient’s age and serum AMH level.

Furthermore, we investigated the association between the serum AMH level and cumulative live birth rate. Women with a high serum AMH level had a higher cumulative live birth rate and shorter treatment period than women with a low serum AMH level. These results were similar to those observed in COS IVF cycles [49, 50]. However, some previous studies showed that basal AMH has modest predictive performance for the occurrence of cumulative live birth rate and may not give additional value to the patient age [51, 52]; therefore, studies on the correlation between serum AMH and cumulative live birth rate have inconsistent findings even in COS cycles. To validate our findings, the correlation of the serum AMH level with cumulative live birth rate in CC-based minimal stimulation should be further evaluated.

The strength of this study is the assessment of a relatively rare cohort in the current IVF practice. Specifically, given that very few clinics use a CC-based minimal ovarian stimulation protocol, our findings provide useful insights for the improvement of clinical outcomes in CC-based minimal stimulation IVF. However, considering the single-center, retrospective design of our study, the possibility of selection biases cannot be excluded and therefore our results are limited. Further prospective, large-scaled studies are warranted to investigate the prediction value of AMH levels in CC-based minimal ovarian stimulation.

## Conclusions

The serum AMH level is positively correlated with ovarian responsiveness, embryo developmental competence during the pre-implantation period, cumulative live birth rate, and treatment period in CC-based minimal ovarian stimulation IVF. Therefore, the cycle success rate can be predicted by measuring the serum AMH level before the initiation of the first fertility treatment using minimal ovarian stimulation with CC alone.

## Supplementary Information

**Additional file 1: Table S1.** Cohort characteristics. **Table S2.** Patient characteristics stratified by the serum AMH level. **Table S3.** IVF outcomes of the first cycles, stratified by the serum AMH level. **Table S4.** Pregnancy outcomes of the first cycle stratified by the serum AMH level. **Table S5.** Pregnancy outcomes of the first cycle stratified by the serum AMH level. **Table S6.** Adjusted odds ratio for the blastocyst cryopreservation/live birth rate in the first cycle. **Table S7.** Hazard ratio for cumulative live birth rate per treatment period. **Table S8.** Cumulative live birth rates in the first IVF cycles.

## Data Availability

The data sets used and/or analyzed during the present study are available from the corresponding author on reasonable request.

## Abbreviations

AMH: Anti-Müllerian hormone
CC: Clomiphene citrate
cIVF: Conventional in vitro fertilization
COCs: Cumulus-oocyte complexes
COS: Controlled ovarian stimulation
FSH: Follicle stimulating hormone
GnRH: Gonadotropin-releasing hormone
HTF: Human tubal fluid
ICSI: Intracytoplasmic sperm injection
IVF: In vitro fertilization
LH: Luteinizing hormone
ROC: Receiver operating characteristic
