# Supplementary material for: Anti-Müllerian hormone is correlated with cumulative live birth in minimal ovarian stimulation with clomiphene citrate: a retrospective cohort study
Source: BMC Pregnancy Childbirth. 2020 Nov 27;20:740. doi: 10.1186/s12884-020-03446-1 (PMC7694423; doi:10.1186/s12884-020-03446-1)
Supplement: Supplementary file 1 — Additional file 1: Table S1. Cohort characteristics. Table S2. Patient characteristics stratified by the serum AMH level. Table S3. IVF outcomes of the first cycles, stratified by the serum AMH level. Table S4. Pregnancy outcomes of the first cycle stratified by the serum AMH level. Table S5. Pregnancy outcomes of the first cycle stratified by the serum AMH level. Table S6. Adjusted odds ratio for the blastocyst cryopreservation/live birth rate in the first cycle. Table S7. Hazard ratio for cumulative live birth rate per treatment period. Table S8. Cumulative live birth rates in the first IVF cycles. [file 12884_2020_3446_MOESM1_ESM.docx]

Supplemental Table 1. Cohort characteristics

| No. of patients, n | 1,132 |
| --- | --- |
| Female age, Mean ± SD (range) | 37.1 ± 4.2 (25–46) |
| Male age, Mean ± SD (range) | 39.4 ± 5.8 (25–60) |
| Ovarian stimulation, n (%) |  |
| Natural | 66 (5.8) |
| Letrozole | 53 (4.7) |
| Clomiphene citrate | 689 (60.9) |
| Clomiphene citrate with human menopausal gonadotrophin | 42 (3.7) |
| Clomiphene citrate with letrozole | 42 (3.7) |
| Combination | 240 (21.2) |
| Infertility Cause, n (%) |  |
| Ovulation factor | 43 (3.8) |
| Oviduct factor | 73 (6.4) |
| Endometrial factor | 94 (8.3) |
| Male factor | 142 (12.5) |
| Combination | 48 (4.2) |
| Unexplained | 732 (64.7) |

Supplemental Table 2. Patient characteristics stratified by the serum AMH level

|  | AMH (< 1.42 ng/mL) | AMH (≥ 1.42 ng/mL) | P value |
| --- | --- | --- | --- |
| No. of patients, n | 294 | 395 |  |
| Female age, mean ± SD (range) | 39.7 ± 3.3 (29–46) | 37.7 ± 3.2 (27–46) | < 0.0001 |
| Male age, mean ± SD (range) | 41.7 ± 5.3 (29–59) | 39.8 ± 5.5 (26–60) | < 0.0001 |
| Serum AMH level, mean ± SD (range) | 0.78 ± 0.38 (0.05–1.41) | 3.00 ± 1.66 (1.42–9.51) | < 0.0001 |
| Infertility cause, n (%) |  |  | 0.5095 |
| Ovulation factor | 1 (0.3) | 6 (1.5) |  |
| Oviduct factor | 17 (5.8) | 32 (8.1) |  |
| Endometrial factor | 24 (8.2) | 33 (8.4) |  |
| Male factor | 43 (14.6) | 49 (12.4) |  |
| Combination | 13 (4.4) | 18 (4.6) |  |
| Unexplained | 196 (66.7) | 257 (65.1) |  |

AMH, anti-Müllerian hormone

Supplemental Table 3. IVF outcomes of the first cycles, stratified by the serum AMH level

|  | AMH (< 1.42 ng/mL) | AMH (≥ 1.42 ng/mL) | P value |
| --- | --- | --- | --- |
| No. of oocyte retrieval cycles | 294 | 395 |  |
| No. of successful oocyte retrieval cycles (≥ 1 oocyte), n (%) | 256 (87.1) | 372 (94.2) | 0.0071 |
| No. of antral follicles, mean ± SD | 4.82 ± 2.49 | 10.22 ± 5.58 | <0.0001 |
| No. of fully developed antral follicles, Mean ± SD | 2.85 ± 1.34 | 3.09 ± 1.65 | 0.0365 |
| No. of retrieved oocytes, mean ± SD | 1.81 ± 1.27 | 2.15 ± 1.30 | 0.0005 |
| Oocyte collection rate, mean ± SD (%, per an aspirated follicle) | 73.7 ± 34.1 | 79.3 ± 29.9 | 0.0416 |
| No. of fertilized oocytes, mean ± SD | 1.33 ± 1.12 | 1.72 ± 1.21 | < 0.0001 |
| Fertilization rate, mean ± SD (%) | 75.0 ± 35.6 | 79.2 ± 32.4 | 0.0503 |
| No. of cleaved embryos, mean ± SD | 1.30 ± 1.11 | 1.68 ± 1.21 | < 0.0001 |
| Cleavage rate, mean ± SD (%) | 97.3 ± 13.5 | 97.3 ± 14.4 | 0.9614 |
| Morphological grade on day 2, based on Veeck’s criteria |  |  | 0.0030 |
| Grade 1, n (%) | 42 (11.0)^a^ | 127 (19.1)^b^ |  |
| Grade 2, n (%) | 118 (30.9) | 207 (31.1) |  |
| Grade 3, n (%) | 208 (54.5)^a^ | 316 (47.5)^b^ |  |
| Grade 4, n (%) | 14 (3.7) | 15 (2.3) |  |
| No. of embryos cultured to the blastocyst stage, mean ± SD | 0.77 ± 0.97 | 1.04 ± 1.09 | 0.0008 |
| No. of blastocysts, mean ± SD | 0.50 ± 0.76 | 0.80 ± 0.98 | <0.0001 |
| Blastocyst formation rate, mean ± SD (%) | 66.2 ± 42.6 | 75.5 ± 38.1 | 0.0276 |
| No. of cryopreserved blastocysts, mean ± SD | 0.44 ± 0.71 | 0.75 ± 0.94 | <0.0001 |
| Blastocyst cryopreservation rate, mean ± SD (%) | 57.1 ± 44.6 | 70.6 ± 40.2 | 0.0024 |
| Morphological grade of inner cell mass |  |  | 0.0681 |
| Grade A, n (%) | 31 (24.0) | 51 (17.2) |  |
| Grade B, n (%) | 41 (31.8) | 127 (42.9) |  |
| Grade C, n (%) | 57 (44.2) | 118 (39.9) |  |
| Morphological grade of trophectoderm |  |  | 0.9018 |
| Grade A, n (%) | 19 (14.7) | 43 (14.5) |  |
| Grade B, n (%) | 36 (27.9) | 89 (30.1) |  |
| Grade C, n (%) | 74 (57.4) | 164 (55.4) |  |

AMH, anti-Müllerian hormone; IVF, in vitro fertilization

Supplemental Table 4. Pregnancy outcomes of the first cycle stratified by the serum AMH level

|  | AMH (< 1.42 ng/mL) | AMH (≥ 1.42 ng/mL) | P value |
| --- | --- | --- | --- |
| No. of patients who received embryo transfer, n (%) | 183 (62.2) | 293 (74.2) | 0.0026 |
| No. of embryo transfer cycles | 236 | 415 |  |
| Fresh: cleavage stage embryo |  |  |  |
| Transferred embryo, n | 80 | 174 |  |
| Endometrial thickness (mm), mean ± SD | 10.7 ± 2.2 | 10.8 ± 2.3 | 0.9022 |
| Clinical pregnancy, n, (%) | 19 (23.8) | 80 (46.0) | 0.0007 |
| Ongoing pregnancy, n, (%) | 15 (18.8) | 67 (38.5) | 0.0018 |
| Follow-up data on live birth* | 80 | 174 |  |
| Live birth, n, (%)* | 14 (17.5) | 64 (36.8) | 0.0020 |
| Frozen: cleavage stage embryo |  |  |  |
| Transferred embryo, n | 58 | 48 |  |
| Endometrial thickness (mm), mean ± SD | 9.9 ± 1.1 | 9.9 ± 1.4 | 0.9637 |
| Clinical pregnancy, n, (%) | 16 (27.6) | 27 (56.3) | 0.0028 |
| Ongoing pregnancy, n, (%) | 13 (22.4) | 23 (47.9) | 0.0058 |
| Follow-up data on live birth* | 53 | 43 |  |
| Live birth, n, (%)* | 12 (22.6) | 19 (44.2) | 0.0248 |
| Frozen: blastocyst stage embryo |  |  |  |
| Transferred embryo, n | 98 | 193 |  |
| Endometrial thickness (mm), mean ± SD | 10.3 ± 2.2 | 10.7 ± 2.2 | 0.6895 |
| Clinical pregnancy, n, (%) | 51 (52.0) | 105 (54.4) | 0.7025 |
| Ongoing pregnancy, n, (%) | 47 (48.0) | 77 (47.2) | 0.8961 |
| Follow-up data on live birth* | 92 | 177 |  |
| Live birth, n, (%)* | 34 (37.0) | 73(41.2) | 0.4956 |
| Clinical pregnancy in the first IVF cycles, n, (%) | 83 (28.2) | 198 (50.1) | < 0.0001 |
| Ongoing pregnancy in the first IVF cycles, n, (%) | 72 (24.5) | 144 (44.6) | < 0.0001 |
| Live birth in the first IVF cycles, n, (%) | 60 (20.4) | 156 (39.5) | < 0.0001 |

*ET date: November 2017–December 2018

AMH, anti-Müllerian hormone; IVF, in vitro fertilization

Supplemental Table 5. Pregnancy outcomes of the first cycle stratified by the serum AMH level

|  | AMH (ng/mL), stratified by the percentile | | | | P value |
| --- | --- | --- | --- | --- | --- |
|  | < 0.87 | 0.87 ≤, < 1.59 | 1.59 ≤, < 2.70 | 2.70 ≤ |  |
| No. of patients | 173 | 169 | 174 | 173 |  |
| No. of patients who received embryo transfer, n (%) | 101 (58.4)^a^ | 115 (68.1)^b^ | 127 (73.0)^b^ | 133 (76.9)^b^ | 0.0014 |
| No. of embryo transfer cycles | 132 | 148 | 191 | 180 |  |
| Fresh: cleavage stage embryo |  |  |  |  |  |
| Transferred embryo, n | 44 | 55 | 81 | 74 |  |
| Clinical pregnancy, n, (%) | 5 (11.4)^a^ | 23 (41.8)^b, c^ | 31 (38.3)^b^ | 40 (54.1)^c^ | < 0.0001 |
| Ongoing pregnancy, n, (%) | 4 (9.1)^a^ | 18 (32.7)^b, c^ | 24 (29.6)^b^ | 36 (48.7)^c^ | 0.0002 |
| Follow-up data on live birth* | 44 | 55 | 81 | 74 |  |
| Live birth, n, (%)* | 4 (9.1)^a^ | 17 (30.9)^b, c^ | 23 (28.4)^b^ | 34 (46.0)^c^ | 0.0005 |
| Frozen: cleavage stage embryo |  |  |  |  |  |
| Transferred embryo, n | 30 | 36 | 21 | 19 |  |
| Clinical pregnancy, n, (%) | 7 (23.3) | 15 (41.7) | 10 (47.6) | 11 (57.9) | 0.0892 |
| Ongoing pregnancy, n, (%) | 6 (20.0) | 12 (33.3) | 10 (47.6) | 8 (42.1) | 0.1776 |
| Follow-up data on live birth* | 27 | 34 | 18 | 17 |  |
| Live birth, n, (%)* | 5 (18.5) | 12 (35.3) | 8 (44.4) | 6 (35.3) | 0.2875 |
| Frozen: blastocyst stage embryo |  |  |  |  |  |
| Transferred embryo, n | 58 | 57 | 89 | 87 |  |
| Clinical pregnancy, n, (%) | 28 (48.3) | 32 (56.1) | 45 (50.6) | 51 (58.6) | 0.5680 |
| Ongoing pregnancy, n, (%) | 25 (43.1) | 30 (52.6) | 38 (42.7) | 45 (51.7) | 0.4758 |
| Follow-up data on live birth* | 55 | 53 | 80 | 81 |  |
| Live birth, n, (%)* | 19 (34.6) | 22 (41.5) | 27 (33.8) | 39 (48.2) | 0.2330 |
| Clinical pregnancy in the first IVF cycles, n, (%) | 38 (22.0)^a^ | 67 (39.6)^b^ | 83 (47.7)^b, c^ | 93 (53.8)^c^ | < 0.0001 |
| Ongoing pregnancy in the first IVF cycles, n, (%) | 33 (19.1)^a^ | 58 (34.3)^b^ | 73 (42.0)^b, c^ | 84 (48.6)^c^ | < 0.0001 |
| Live birth in the first IVF cycles, n, (%) | 28 (16.2)^a^ | 51 (30.2)^b^ | 61 (35.1)^b, c^ | 76 (43.9)^c^ | < 0.0001 |

*ET date: November 2017–December 2018

AMH, anti-Müllerian hormone; IVF, in vitro fertilization

Supplemental Table 6. Adjusted odds ratio for the blastocyst cryopreservation/live birth rate in the first cycle

|  | Adjusted odds ratio | 95% confidential intervals | P value | AUC |
| --- | --- | --- | --- | --- |
| **Blastocyst cryopreservation rate*** |  |  |  | 0.597 |
| AMH (< 1.42 ng/mL) | Reference | – | – |  |
| AMH (≥ 1.42 ng/mL) | 1.778 | 1.249–2.532 | 0.0014 |  |
| **Live birth rate**** |  |  |  |  |
| Fresh: cleavage stage embryo*** |  |  |  | 0.667 |
| AMH (< 1.42 ng/mL) | Reference | – | – |  |
| AMH (≥ 1.42 ng/mL) | 1.840 | 1.040–3.386 | 0.0351 |  |
| Frozen: cleavage stage embryo**** |  |  |  | 0.691 |
| AMH (< 1.42 ng/mL) | Reference | – | – |  |
| AMH (≥ 1.42 ng/mL) | 1.699 | 0.698–4.180 | 0.2423 |  |
| Frozen: blastocyst stage embryo***** |  |  |  | 0.681 |
| AMH (< 1.42 ng/mL) | Reference | – | – |  |
| AMH (≥ 1.42 ng/mL) | 1.563 | 0.926–2.662 | 0.0948 |  |

* Confounding factors: female age, male age. The developmental outcomes of 635 embryos were used for this analysis.

** Confounding factors: female age, male age, endometrial thickness.

*** The clinical outcomes in 254 cycles were used for this analysis.

**** The clinical outcomes in 106 cycles were used for this analysis.

***** The clinical outcomes in 291 cycles were used for this analysis.

AMH, anti-Müllerian hormone; AUC, area under the curve

Supplemental Table 7. Hazard ratio for cumulative live birth rate per treatment period

|  | Unadjusted hazard ratio (95% CI) | P-value |  | Adjusted hazard ratio (95% CI) | P-value |
| --- | --- | --- | --- | --- | --- |
| Female age | 0.886 (0.860–0.914) | < 0.0001 |  | 0.887 (0.852–0.923) | < 0.0001 |
| Male age | 0.959 (0.938–0.979) | 0.0001 |  | 1.011 (0.985–1.037) | 0.4106 |
| AMH** | 1.159 (1.094–1.224) | < 0.0001 |  | 1.104 (1.036–1.171) | 0.0015 |

AMH, anti-Müllerian hormone; CI, confidence interval

*The clinical outcomes of 689 patients were used for this analysis.

**The serum AMH value was used as the continuous parameter in this analysis.

Supplemental Table 8. Cumulative live birth rates in the first IVF cycles

|  | AMH (ng/mL), stratified by the percentile | | | | *P* value |
| --- | --- | --- | --- | --- | --- |
|  | < 0.87 | 0.87 ≤, < 1.59 | 1.59 ≤, < 2.70 | 2.70 ≤ |  |
| Female age < 38 years | 13/34 (38.2) | 20/55 (36.4) | 29/68 (42.7) | 47/84 (56.0) | 0.0223 |
| Female age 38–40 years | 8/54 (14.8) | 21/62 (33.9) | 25/73 (34.3) | 22/60 (36.7) | 0.0177 |
| Female age > 40 years | 7/85 (8.2) | 10/52 (19.2) | 7/33 (21.2) | 7/29 (24.1) | 0.0185 |
| *P* value | 0.0001 | 0.0554 | 0.0354 | 0.0011 |  |

AMH, anti-Müllerian hormone; IVF, in vitro fertilization
